# Vegetables: New Zealand Children Are Not Eating Enough

**DOI:** 10.3389/fnut.2018.00134

**Published:** 2019-01-08

**Authors:** Elaine Rush, Fa'asisila Savila, Shabnam Jalili-Moghaddam, Isaac Amoah

**Affiliations:** ^1^Child Health Research Centre, Auckland University of Technology, Auckland, New Zealand; ^2^Riddet Institute, Massey University, Palmerston North, New Zealand; ^3^Centre for Pacific Health and Development Research, Auckland University of Technology, Auckland, New Zealand

**Keywords:** vegetables, lifecourse health, supply, cost, children, actions

## Abstract

We know that eating a variety of vegetables every day is associated with favorable health across the lifecourse. Internationally, food-based dietary guidelines encourage the consumption of a variety of vegetables and fruit but globally,people are not eating enough vegetables to meet the three-or-more-a-day guideline. Vegetables are good sources of vitamins and minerals, fiber, and many bioactive compounds that promote health and provide energy. They also help reduce hidden hunger (micronutrient deficiencies) and support the healthy growth and development of children. New Zealand is a world leader in the production of diverse nutrients and foods yet poverty and other environmental barriers mean only one in two children eats three-or-more servings of vegetables a day. Price and availability are limiting factors. The proliferation of community, school and home vegetable gardens and vegetable cooperatives may improve access. On a macro level, upstream policies such as a “living wage,” affordable housing, and land-use planning are required. International dietary solutions include an agricultural shift to intensified horticulture with a focus on vegetables. The consumption of more plant-based foods including vegetables would reduce green-house gases, reduce land clearing, and help prevent diet-related disease if consumed daily across the lifecourse.

## Introduction

### Lifecourse Health—Plenty of Vegetables Every Day of Life

Recommendations that vegetables and fruit should be consumed every day, from the time solid food starts to complement breast milk, is based on convincing evidence of favorable health, growth, and development associated with their intake (1). For children, this includes the prevention of cardio-metabolic disease (2) and obesity (3). Vegetables are one important part of a lifelong dietary pattern that is lower in energy density and higher in nutrient density. Specifically, vegetables contribute essential fiber, vitamins and minerals and many health-promoting bioactive compounds. Replacing energy-dense foods with more water-rich vegetables (4) not only lowers energy density but increases the volume of food ingested and satiety while improving overall dietary pattern. By volume it is recommended that a variety of colored vegetables make up more than one quarter of all food consumed each day and this is the basis for the MyPlate communication tool (5).

### Food-Based Dietary Guidelines

Globally, food-based dietary guidelines (FBDGs, rather than nutrient-based) encourage the consumption of a variety of vegetables and fruit (6) as part of a dietary pattern that provides the required nutrients to the general public. The focus of FBDGs are on food groups rather than nutrients and they emphasize the need to select a variety of foods within each food group. Foods are grouped by their nutrient profile. For example, there is a focus on foods grouped for their protein content such as fish, lean meat, soy products, eggs, nuts, seeds, and legumes. Another group of mainly carbohydrate foods such as rice, wheat, maize and potatoes make up a large portion of the global energy supply. For some populations, foods that contain calcium such as dairy and bones in fish are a focus. Finally, the food group with arguably the biggest variety, vegetables and fruit, receives much attention in guidelines (Table 1) and universal messages which emphasize eating more. Note that the term used currently is vegetables and fruit rather than fruit and vegetables to indicate the importance of vegetables and, now, the recognition that the most nutrient-dense part of a vegetable or fruit is next to the skin (7).

**Table 1:** Food-based dietary guidelines relating to vegetables and fruit from selected countries.

**Country**	**Message**
Sweden	More vegetables and fruit - Eat lots of fruit, vegetables and berries! Ideally, choose high fiber vegs such as root vegetables, cabbage, cauliflower, broccoli, beans and onions.
South Africa	Eat plenty of vegetables and fruit every day.
Mexico	Include vegetables and fresh fruits in each meal. Choose them with peel and in season.
United States	A variety of vegetables from all of the subgroups-dark green, red and orange, legumes (beans and peas), starchy, and others
New Zealand	Eat plenty of vegetables and fruits.
Australia	Plenty of vegetables, including different types and colors, and legumes/beans
Iran	Eat raw and cooked vegetables every day at main meals and snacks
India	Eat plenty of vegetables and fruits
China	Consume plenty of vegetables, milk, and soybeans

Food-based dietary guidelines reported by the FAO (6)

### Why Are Vegetables Healthy?

Vegetables are healthy if a variety are consumed as together they provide diverse combinations of essential nutrients and dietary fiber. The dietary diversity score devised by the FAO (8) is one proxy measure of nutrient adequacy and identifies 16 groups of foods. Four of these groups describe vegetable diversity and the importance of each group for diverse and essential nutrients. These four groups are 1. White tubers and roots which are good sources of energy and fiber; 2. “vitamin A rich vegetables and tubers: pumpkin, carrot, squash, or sweet potato that are orange inside, and other locally available vitamin A rich vegetables (e.g., red sweet pepper)”; 3. “dark green leafy vegetables, including wild forms and locally available, vitamin A rich leaves such as amaranth, cassava leaves, kale and spinach” which are also good sources of folate; 4. “other vegetables (e.g., tomato, onion, eggplant) and other locally available vegetables.” A higher dietary diversity score is associated with improved growth in children (9) and micronutrient status (10). Vegetables are good sources of the essential nutrients, dietary fiber, vitamins, and minerals. Dietary fiber, broadly described as carbohydrates that are not digested in the small intestine, has benefits to health associated with modifying the rate of transit through the bowel and delaying the absorption of nutrients such as glucose, binding, and excretion of bile acids, fermentation in the large intestine, and the production of butyrate and adding to stool bulk and improving laxation (11). Vegetables are good sources of viscous and non-viscous fibers and diets higher in fiber has been linked to cancer prevention, weight management, lower risk of heart disease and diabetes management and prevention (12).

In addition, beneficial health effects are derived from bioactive compounds such as phenolics, flavonols, flavonoids, capsaicinoids and carotenoids which are found for example in spinach, broccoli, lettuce, onions, pepper, and tomatoes.

There is a renewed focus on hidden hunger which describes micronutrient deficiencies (13) which may be present with obesity, another form of malnutrition. In children this will impact on growth, and development. Whole vegetables and fruits not only enhance consumers' bioactive stores (14) but also improve micronutrient status. Thus, there is a synergistic impact on health derived from the combination of vitamins, minerals, flavonoids, phenolics, pigments, peptides polysaccharides, and fibre (soluble and insoluble) because of the consumption of whole or minimally processed vegetables. Prebiotics such as the oligosaccharides inulin and fructo-oligosaccharides (15) found in vegetables and fruits have been shown to stimulate growth of probiotic bacteria (bifidobacteria and lactic acid bacteria) in the colon which favorably enhances the function of the gastrointestinal and immune systems (16). This unique property of prebiotics is attributed to their ability to resist digestion in the small intestine, eventually undergoing fermentation in the colon. In addition, prebiotics have been shown to increase the absorption of calcium and magnesium, influence blood glucose levels, and improve plasma lipids (16) and laxation.

The need to promote the consumption of vegetables and other plant-based foods compared to animal-based products, such as beef, is warranted due to the more favorable impact of plant rather than animal agriculture on the planet (17). For example, to raise cattle, large quantities of water are required both to produce the grass or feed for the cattle and for the cattle to drink. Cattle emit green-house gases and may pollute the water system. In contrast, vegetable cultivation requires a relatively small amount of water, could be done without pesticides, absorbs carbon dioxide from the atmosphere, and produces oxygen. An intensification of vegetable production is a sustainable way to support populations to meet dietary guidelines, reduce food insecurity, improve health and provide some amelioration to adverse climate change (18).

### Consumption—Are Children Eating Their Vegetables?

Worldwide the consumption of fruit and vegetables is low (19) and dependent on household income. Miller and colleagues calculated, with data from 18 countries and 143,305 participants, that in low-income countries three servings of vegetables and two servings of fruit each day for each household member would cost 52% of the household income while in upper-middle income countries the cost was 2%. Vegetables and fruits were more expensive for rural households and consumption decreased as cost increased.

### NZ as a Case Study

New Zealand is a world leader in the production of diverse nutrients and foods (20). In New Zealand from 2012 to 2017 the hectares devoted to growing onions increased by 5% but potatoes, buttercup/squash, peas and sweetcorn reduced by 18, 15, 40, and 17%. The number of dairy cattle increased 1%. Currently, 27% of New Zealand children are living in income poverty and 12% of children are living in material hardship which means among other factors reduced access to vegetables and fruit and/or going to school hungry. Food security which includes having time, money and resources to access and prepare food is a substantial and complex problem. From a study that measured the relative change in price of healthier and less healthy foods over 10 years in New Zealand, the authors reported that the price of wholesome healthy foods and minimally processed foods were cheaper in summer compared to less healthy foods and processed foods (21). At the same time the vegetable price index rose from 700 to 900 between 2006 and 2018- an increase of 35%; fruit rose 60% in the same time (22). In the same period, bread and cereals rose 19% and overall the food price index rose 33%.

Over the last 16 years only slightly more than half the children in New Zealand are consuming 3 or more serves of vegetables each day (Table 2). At the same time, more than half are consuming sugary drinks and fast foods at least once a week (24). Some groups, such as Pacific Island children, are eating less vegetables than the general population of children. In New Zealand, the most commonly consumed vegetables are potato, potato fries, kumara (sweet potato), carrots, broccoli, peas, lettuce, cauliflower, cabbage, tomatoes, and corn(23). Onions are frequently added to recipes (30). Overall, approximately 25% of the daily dietary fiber intake (median 19g/day) is derived from vegetables (23).

**Table 2:** Proportions of New Zealand children meeting the vegetable guideline of three or more serves of vegetables a day.

**Year**	**Age, y**	**% meeting vegetable guideline**	**References**
**NATIONAL SURVEYS**			
2002	5-14	57%	(23)
2011-2014	0 to 14	57%	(24)
2014-2017	0 to 14	53%	(24)
**METFORMIN IN GESTATIONAL DIABETES FOLLOW UP STUDY**			
2008	2	76 % (*n* = 147)	(25)
2014	7 to 9	37% (*n* = 99)	Unpublished data
**PACIFIC ISLANDS FAMILIES STUDY**			
2004	4	35% (*n* = 739)	(26)
2006	6	35% (*n* = 646)	(27)
2009	9.4	49% (*n* = 976)^*^	(28)
2014	14	32% (*n* = 931)	Unpublished data

NB, Adequate vegetable intake is defined for children aged 2–4 years as eating at least two servings of vegetables each day and for children aged 5–14 years as eating at least three servings of vegetables each day (29)—all surveys were food frequency questionnaires except for 2009^*^ which was a dietary habits questionnaire. CNS Children's Nutrition Survey; MIGTOFU Metformin in gestational diabetes: the follow-up study; PIF Pacific Islands Families study.

Children depend on their parents and caregivers for food and in low income families there is a tendency to buy more energy dense food, to get the most calories for the money, rather than nutrient dense foods such as vegetables and fruit (31). Food, especially healthier food, is the first essential that low income families compromise on in times of hardship, exacerbating existing nutritional deficiencies resulting from general lack of money (32). As children grow, the overall volume of food intake increases but there is a fall in the quantity of fruit and vegetables and an increase in other foods such as—energy-dense and low cost breakfast cereal and/or convenience foods. We demonstrated this in the longitudinal Pacific Islands Families study between child ages 4 and 6 years (27). In New Zealand, for families living in more highly deprived areas increases in fruits and vegetable prices especially around their off-season compel them to substitute the purchase of healthier whole fruit and vegetables with cheap energy-dense nutrient-poor products. The weight of vegetables required each week for a family of 6 is 12.6 kg (3 serves × 100g × 7 days × 6 people) and provides one fifth to one third of the energy that uncooked rice or pasta which in addition are more robust to transport and have a longer storage time.

### Health Promotion and the Way Forward

Messages and actions concerning vegetable consumption within New Zealand and globally are consistent but the barriers are persistent. In addition to cost, the perishability and therefore waste of most fresh whole fruits and vegetables discourages purchase. Frozen and canned vegetables may be a better option and should be included more explicitly in health promotion messages. There are other food processing techniques such as chilling, modified atmospheres, edible coatings that enhance the shelf-life of whole vegetables (33) but they add cost and the addition of packaging presents other problems. Drying and freeze drying are also options but require controlled storage conditions.

Locally grown vegetables are the ideal and every city should have dedicated land for market gardens. In addition, community, school and home vegetable gardens and cooperatives are proliferating in New Zealand. New Zealand is fortunate in that it has a temperate climate which allows a continuous source of vegetables throughout the year. In secondary schools (school years 9 to 13), it has been shown that children attending a school with a garden had a lower body mass index and also reported less fast food consumption (34).

More collaboration between industry, retail, government, and not-for-profit organizations promoting public health is required (35). In addition, town planning, land use, reduction in price and increase in availability of vegetables and equitable and culturally targeted actions to improve vegetable intake for children are required. Further, fruits and vegetables could be targeted separately rather than together, and vegetables could be included in cooking education.

To improve conditions on a micro level, upstream policies such as a “living wage” (36), affordable housing, and land-use planning are required. Global dietary solutions, including an agricultural shift to producing more and at the same time consuming more vegetables and plant-based foods throughout the lifecourse, would reduce green-house gases, reduce land clearing, and help prevent diet-related disease. This is a global challenge.

## Author Contributions

ER led the conception and writing of the manuscript, SJ-M, FS, and IA contributed and critically revised the manuscript. All authors approved the final manuscript.

### Conflict of Interest Statement

The authors declare that the research was conducted in the absence of any commercial or financial relationships that could be construed as a potential conflict of interest.
